# Complexity and potentials of clinical feedback in mental health: an in-depth study of patient processes

**DOI:** 10.1007/s11136-020-02550-1

**Published:** 2020-06-15

**Authors:** Stig Magne Solstad, Gøril Solberg Kleiven, Christian Moltu

**Affiliations:** 1 District General Hospital of Førde, Førde, Norway; 2 District General Hospital of Førde & Department of Health and Caring Science, Western Norway University of Applied Science, Førde, Norway

**Keywords:** Routine outcome monitoring, Clinical feedback systems, Qualitative research

## Abstract

**Purpose:**

Routine outcome monitoring (ROM) and clinical feedback systems (CFS) are becoming increasingly prevalent in mental health services. Their overall efficacy is unclear, but quantitative evidence suggests they can be useful tools for preventing treatment failure and enhancing therapeutic outcomes, especially for patients who are not progressing in therapy. The body of qualitative material, however, is smaller and less refined. We need to know more about how ROM/CFS is used in psychotherapy, and why it is helpful for some patients, but not others.

**Methods:**

We recorded therapy sessions of 12 patients who were using a CFS as part of their therapies at an outpatient clinic in Norway. We then conducted video-assisted interviews and follow-up interviews with patients. Data were analyzed with systematic text condensation.

**Results:**

Analysis revealed three themes: (1) triggering reflections, emotions, and self-awareness, (2) Ambivalent and ambiguous self-presentation, and (3) potential for feeling understood and talking about what matters.

**Conclusion:**

Answering questions in a CFS is an interpretative and intentional process of self-presentation and the results from ROM/CFS must be interpreted and explored in conversation to be clinically useful. When they are, they have potential for enhancing the therapeutic process by stimulating self-awareness, reflexivity, and allowing access to new therapeutic topics. Further research should explore this how-to aspect of ROM/CFS with different CFS and different types of patients. Integrating clinical feedback in therapeutic practice can be conceptualized as a clinical skill, which should be a part of training programs for therapists.

**Electronic supplementary material:**

The online version of this article (10.1007/s11136-020-02550-1) contains supplementary material, which is available to authorized users.

## Introduction

Routine outcome monitoring (ROM) is the process of routinely collecting data about patients to measure their progress. In mental health services, these outcomes are increasingly being measured by clinical feedback systems (CFS). A CFS is usually based on self-report and consists of a standardized set of items about parameters relevant for mental health treatment. An estimated 60–65% of patients in psychotherapy show significant positive change in therapy, but 30–35% show no change and 5–10% deteriorate [1, 2]. ROM/CFS provides feedback to therapists about patients’ progress, allowing therapists to improve their skills and adjust their approaches to patients who are not progressing [3, 4]. Several CFS have been developed in the past decades, and ROM/CFS practices are becoming increasingly prevalent in the US, Australia, New Zealand, and Europe [5, 6]. Meta-analyses of the efficacy of ROM have given mixed results, but it seems clear that using ROM/CFS can enhance psychological therapies, especially for patients who are not progressing, or deteriorating, in therapy [7–9]. There is evidence that therapist commitment to using ROM/CFS has a positive effect on patient’s rates of change [10], and that feedback is more effective when delivered to both therapists and patients [11]. However, we need to know more about why ROM/CFS is helpful for some, but not all, patients. Qualitative inquiries into patient perspectives hold great promise in this regard.

A recent qualitative metasynthesis found 16 studies concerning the patient perspective [12]. The authors found four common themes. (1) Patients showed some suspicion toward service providers about how the information from ROM/CFS would be used. For example, some patients feared the results from the CFS may be used by service providers to take unfair credit for progress, or deny further access to services. (2) ROM/CFS may not capture the complexity of patients’ lives. A common complaint was that CFS measured symptoms, but neglected goals, values, and positive aspects of life. Patients also reported difficulties with fitting their experiences into predefined categories. Flexibility and support from clinicians was needed to make ROM/CFS useful. (3) ROM/CFS appeared to have a potential for empowering patients. Many patients wanted to be involved in the treatment planning process, and in defining their outcomes. ROM/CFS seemed to facilitate this, when it was used in a trusting therapeutic relationship. (4) ROM/CFS also appeared to have a potential for facilitating collaborative practice, for example by helping to identify therapeutic topics, focusing and structuring sessions, and stimulating reflection and self-awareness. A subsequent qualitative study reported largely similar themes [13].

These findings provide more insight into how ROM/CFS can be applied successfully. However, all 17 studies used some form of retrospective interviewing. They may have captured post hoc attitudes and beliefs rather than experiences. Furthermore, few of them attempted to understand ROM/CFS as a therapeutic process. In this study, we wished to address this gap in the research literature. We explored the following research question: “What are patients’ experiences with routine outcome monitoring and answering clinical feedback systems in mental health services?”

## Method and materials

### CFS context—Norse Feedback

**Fig. 1:**
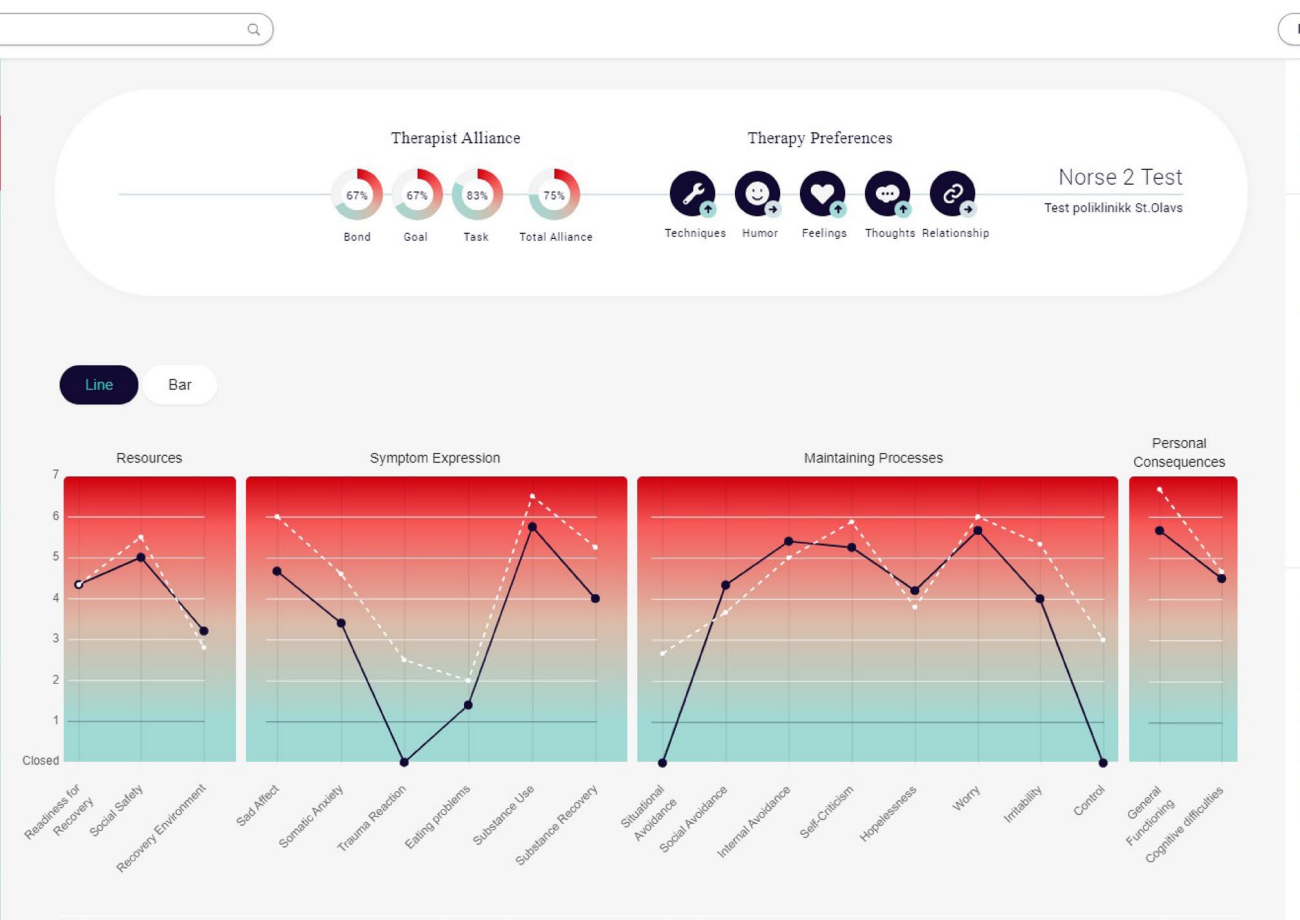
NF report

Norse Feedback (NF) [14, 15] is a CFS developed by the Helse Førde Hospital Trust and standardized for the Norwegian population [16, 17]. Items were generated from clinicians’ and patients’ needs [14], and are tested and refined in clinical implementation studies [18, 19]. The system is administered electronically via PC, computer tablet, or smartphone. In a standard schedule, patients will answer the NF in advance of each session, typically weekly or biweekly. NF currently consists of a maximum total of 99 items loading onto multiple scales: common psychiatric symptoms, alcohol and drug abuse, medications, social and personal functioning, strengths and resources, therapeutic needs, therapeutic progress, and therapeutic alliance. Patients respond to the items on a seven-point Likert scale. The NF generates a visual report that summarizes the patient’s development throughout treatment (Fig. 1). Additionally, therapists can access the full list of patients’ responses to each item. These reports are available to the therapist immediately after the questionnaire is completed, but not to the patient. Therapists may provide feedback to patients about their scores, for example by printing the reports or displaying them on a computer screen. Further information about the development and properties of the NF is provided in the online supplementary material.

### Participants

All patient participants were recruited from a specialist mental health outpatient clinic in Norway, via their therapists. The clinic provides services for patients with moderate to severe impairment, including mood and anxiety disorders, eating disorders, personality disorders, and psychotic disorders. Clinical staff consists of psychiatric residents, psychiatrists, resident psychologists, clinical psychologists, mental health nurses, and social workers. During the recruitment period, a total of 30 therapists worked at the clinic. The mean number of consultations for patients at this clinic was 15.3 (SD = 32.6) in the recruitment period.

The recruitment strategy was naturalistic and by self-selection, aiming for 8–15 participants, per recommendations from consensual qualitative research [20]. The study was presented at staff meetings, and individually to each therapist, via e-mail. All therapists were invited to join as recruiters. Those who did were asked to invite all new eligible patients to join, but they were limited to recruit a maximum of three patients. The inclusion criteria for patients were that they had not already had more than six sessions with their therapist, and were willing to use the NF as part of their therapy. Exclusion criteria were current psychosis or lack of Norwegian language skills necessary to complete the NF. Apart from these criteria, we did not select for participants with regard to gender, ethnicity, or other demographic variables. During the recruitment period, one interested patient was excluded because he had more than six sessions and had never used the NF. The final number of participating patients was 12, recruited from nine therapists. No therapists recruited more than two patients. One patient dropped out of therapy, and the research project, before a follow-up interview could be conducted. Therapist and patient characteristics are provided in Tables 1 and 2. To protect their anonymity, information is presented sparingly. Recruitment took place from February 2017 to December 2018. Interviews took place from June 2017 to February 2019.

**Table 1:** Therapist characteristics

Therapist	Age	Sex	Profession	Years of experience	Therapeutic approach
T1	60s	Female	Mental health nurse	9	Cognitive behavioral therapy
T2	30s	Male	Clinical psychologist	11	Emotion-focused therapy/psychodynamic
T3	20s	Female	Clinical psychologist	2	Cognitive behavioral therapy
T4	30s	Female	Clinical psychologist	3	Psychodynamic/integrative
T5	20s	Female	Clinical psychologist	2	Psychodynamic/integrative
T6	20s	Female	Resident psychologist	1	Emotion-focused therapy/integrative
T7	30s	Female	Clinical psychologist	3	Emotion-focused therapy/integrative
T8	20s	Female	Resident psychologist	1	Cognitive behavioral therapy
T9	40s	Male	Psychiatrist	10	Cognitive behavioral therapy/integrative

**Table 2:** Patient characteristics

Patient	Age	Sex	Interview schedule
P1	30s	Female	After session 6 + 10 weeks later
P2	60s	Female	After session 5 + 16 weeks later
P3	40s	Male	After session 4 + 9 weeks later
P4	40s	Male	After session 7 + 12 weeks later
P5	30s	Male	After session 6 + 12 weeks later
P6	20s	Female	After session 5 + 9 weeks later
P7	20s	Female	After session 7 + 12 weeks later
P8	20s	Female	After session 4 + 11 weeks later
P9	30s	Female	After session 5 + 12 weeks later
P10	20s	Female	After session 3, no follow-up (drop-out)
P11	70s	Female	After session 6 + 9 weeks later
P12	60s	Male	After session 4 + 13 weeks later

### Researchers

All authors are clinical psychologists at the District General Hospital of Førde with six, four, and 12 years of experience, respectively. They have all worked at the outpatient clinic where the data were collected, and are all part of the research group that developed the NF. The first and second authors are PhD candidates of clinical psychology. The last author is a professor of clinical psychology and one of the developers of the NF. He was also the therapist of one of the patient informants. All authors have a general attitude that ROM/CFS can be useful. All authors worked to set their preconceptions aside and approach the data material with an open attitude.

### Data collection procedure

Interpersonal Process Recall (IPR) is an audio- or video-assisted interviewing technique that allows for detailed investigations of interpersonal processes [21, 22] (see the online Supplementary Material). We video recorded one psychotherapy session from each patient participant between sessions 3 and 7. This is a phase where patient and therapist had presumably gotten to know each other and started working with the NF [23, 24].

IPR interviews were conducted within 24 h of the therapy session. After an introduction to the interview method, the interviewer and the participant watched the videos and the participant was asked to stop the playback at events that were relevant to the use of ROM/CFS in psychotherapy. Events were explored using a semistructured interview guide (see the online Supplementary Material). After the videos, patients were asked general questions about their experiences with the NF. To aid recollection, they were given a full list of the items in the NF. To enhance validity, the interviewer continually checked his interpretations of the participants’ statements by summarizing and reformulating the participants’ statements. Two months after the video interview, patients were contacted to schedule a follow-up interview. These interviews were used to check participants’ experiences in retrospect, after they had been consolidated as memories. They did not involve video material, only post-video questions from the original interview guide. Follow-up interviews were conducted between 2 and 4 months after the original interview. Video-assisted interviews varied from 51 to 110 (mean: 82) min in length. Follow-up interviews varied from 33 to 70 (mean: 46) min. All interviews were conducted by the first author.

### Data analysis

**Fig. 2:**
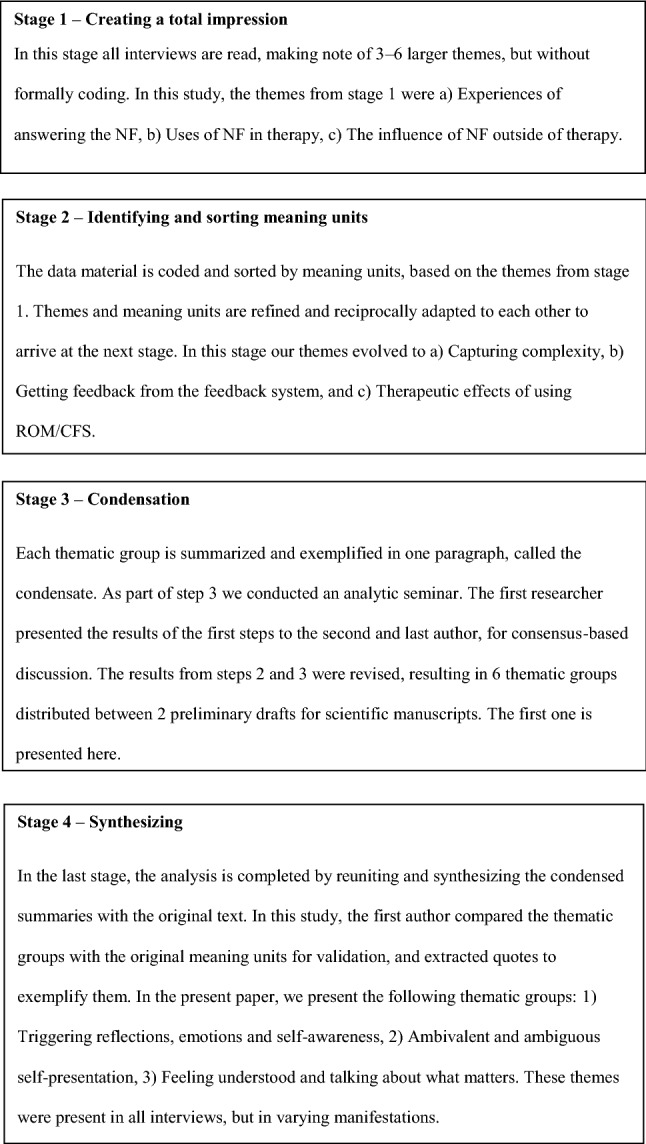
Systematic text condensation analysis

All interviews were transcribed and de-identified by the first author. The transcripts were read by all authors and analyzed using systematic text condensation (STC) [25]. The steps and details of our STC analysis are presented in Fig. 2. The analytic process included a full-day analytic seminar, resulting in two preliminary drafts for scientific papers. The first one, concerning patients’ experiences with answering a CFS, is presented here. The other, concerning patients’ experiences with using ROM/CFS in sessions, is presented elsewhere [26].

### Ethics

This study was approved by the regional ethics committee (REK Sør-Øst; case number 2015/2423) and local data protection officer. Video recordings were stored on secure research servers provided by the research institution. Only the first author had access to the videos. Therapists and patients all received written information about the purpose of the study, confidentiality, anonymity, data storage, and the right to withdraw from the study at any point without any consequences, and all signed forms of written consent when they joined the study.

The interviewer was an experienced clinical psychologist. After all interviews, patient participants were asked about their experiences with the interview and about their present psychological state. None reported excessive distress or other adverse effects of the interview process.

## Results

For the patient participants in this study, from here on referred to as participants, answering a CFS was not undertaken casually or disinterestedly. It was a process of describing and presenting themselves to others that was intentional, motivated, and often conflicted. They wanted to feel understood, but this required more than looking at the report from the CFS. Their answers needed to be elaborated and discussed. When they were, ROM/CFS could make patients feel understood, and guide conversations toward important therapeutic topics. Detailed analyses of these overarching experiences resulted in three core themes: (1) Triggering reflections, emotions, and self-awareness, (2) Ambivalent and ambiguous self-presentation, and (3) Feeling understood and talking about what matters. These themes were present in all interviews, but in varying manifestations. Similar themes emerged from interviews with men and women.

### Triggering reflections, emotions, and self-awareness

Answering feedback items prior to sessions triggered reflections, emotions, and increased self-awareness. Many participants said they became aware of personal and social difficulties, and painful feelings such as sadness, anxiety, hopelessness, and self-criticism. However, several also reported feeling hope, relief, and self-compassion.

I often think that I’m not doing that bad, but when I get these questions and actually evaluate myself, it’s like “Shit, I might be worse off than I thought!”, or “Maybe I’m harsher towards myself than I should be.” For example, regarding suicide. I do think I would be better off dead. I mean, you’re quite hard on yourself when you answer like that (P7)

Though sometimes painful, the process of using a CFS was described as useful and interesting by almost all participants. Most had received feedback about their results, typically by their therapist printing out their reports. As with answering the questions, seeing reports could be demoralizing for some, but also provided helpful self-awareness. Most patients described seeing their own scores and progress as a source of hope and encouragement.

I’m not the type of person that… will boast that “Yes, I’m doing this, now we’re cooking!” I always bring myself down.[…] Norse helps me see that “Aha! This is working!” (P4)

Self-awareness could also go beyond observing status or progression. Many participants said answering the NF gave them more insight into their needs, relationships, and the inevitable ups and downs of life, which was welcome:

The last week… it was very good in the beginning, until we got the terrible message that she was sick […] when I was answering last night, that part was fresher in my mind. But then, I tried to think about what had been good earlier in the week and tried to find a mean value […] So then I realized, it was very good for a while as well. And life is like that, it goes in waves, and that’s normal. (P11)

For this participant, answering the CFS was an important part of therapy. It gave her an understanding of what was painful in her life, but also an appreciation of what was good. For all participants, it seemed that answering the NF helped them, to some degree, understand themselves better.

### Ambivalent and ambiguous self-presentation

Answering a CFS was not a disinterested or objective activity, but a form of self-presentation that demanded interpretation and consideration. Participants were keenly aware that their answers were meant to be read by their therapist. All participants said they wanted their answers to be honest and precise, but most described ambivalence in answering. Many said they were unsure about how to rate their experiences numerically, when to answer, or how to interpret the items. One reason for this was the ambiguity of items:

Here, for example, “I am afraid of losing control with regards to food.” How do you interpret that?

Interviewer: Well, I guess… maybe eating more than you should, or something like that?

Yes, precisely, that’s how I interpret it as well. But I actually scored this highly as well, because I’m afraid of losing control because I don’t eat.[…] I can go 24 hours without eating and lost a lot of weight… 15 kilograms in one month. (P3)

The participant was unsure of how to interpret the item, assuming it was related to binge eating. He scored it highly, hoping that his therapist would bring it up for discussion.

Social desirability also caused ambivalence. Several participants said they were afraid of appearing critical or rude, especially on the items concerning therapeutic preferences. Several also described a fear of being confronted:

Yes, ‘cause I imagine that… I’m struggling a bit with food, like “I need to get treatment”, “No, I don’t need treatment”, and such. And I imagine that if I were to notice, that [I would think] “Now I’ve answered 7 on the topic of food for a long time, I need to start answering 1 soon, or they’ll intervene.” (P9)

Most participants worried that their reports may somehow misrepresent them, leading to misunderstandings or unwanted consequences. Several suggested an optional field for comments in the CFS, to explain themselves:

I would like to express myself more, to elaborate so that I didn’t have to explain it when I came to the session. It would be nice to describe things more and explain, instead of just answering on a scale from 1 to 5. […] Because questions can be interpreted very differently. I can interpret it one way, and you in another way, and then my therapist interprets it in another way. (P8)

Despite participants’ wishes to present themselves precisely and honestly, their answers were affected by their interpretation of items, how they wanted to appear, and what they wanted to communicate to their therapists. Therefore, the reports from the CFS did not always present a correct depiction of their status and needs in therapy.

### Potential for feeling understood and talking about what matters

Despite ambivalence and ambiguities in answering, all participants described experiences with, or seeing potential for, the CFS letting them feel understood and guiding conversations toward important topics. One participant provided a striking example of how answering the CFS led to an important therapeutic insight:

I’m answering the questionnaire based on what people will think of me, not on how I’m really feeling, I’m afraid! But even so, when I’m answering this questionnaire, I don’t ruminate on “what shall I answer,” I answer rather quickly after I’ve read the question, as I feel then and there. But I think it’s quite automatic, that everything’s supposed to be just fine. (P1)

She had been through a rough week, but her symptom scores were low. She struggled to accept her problems, and even more to show other people how she really felt. She avoided this by telling herself, the people around her, and even the CFS, that she was fine. In the session, however, it was clear to the therapist that she was not fine. This disparity allowed for an important exploration of the participant’s way of relating to herself and others.

Many participants reported that using the CFS made them feel like their therapist knew them better, and that it was comforting to know that someone was monitoring their status. For some participants, it was also a way of making sure that important topics would be brought up in the session.

It does make it easier for me. If I were to sit down and talk about everything I felt the past week, then… she really wouldn’t get anything out of me […] I hate that kind of face-to-face, talk-about-feelings [laughter]. I blocked [therapist] totally off when she wanted me to talk about it. So, answering the Norse, that “This is how I feel, this is how I’m doing,” that’s very good because then she knows exactly what to ask to… bring out my feelings.[…] When she’s able to push my buttons with her questions, I get, like, “Now I simply must tell her how I’m feeling.” (P7)

For this participant, the CFS allowed her therapist to hone in on topics that she was not able to verbalize or initiate. For other participants, it was a way of providing a status update, so that sessions could focus on other, underlying therapeutic topics. Several mentioned the items regarding preferences in therapy as particularly useful, because it allowed them more influence and control over their therapies.

Feeling understood and talking about what mattered were what participants wanted from the CFS. Accordingly, feeling misunderstood or talking about irrelevant subjects was what they wanted to avoid. One participant described surprise and frustration when the visual report showed poor normed alliance scores, as she felt her answers had been neutral. She clarified this in the session, but was still upset in the interview. Many participants also expressed frustration because they felt certain items, typically regarding drugs, alcohol, or medications, were irrelevant for them.

It’s annoying! It’s like “What? I don’t have any… I don’t have a drug addiction, why…?” It’s like, you feel insulted, in way. I mean, you don’t really, it’s something [a question] that everybody gets, and everybody’s different, right, but it’s sort of like… [frustrated sigh] […] “Am I afraid that others will hear my thoughts?” and then I feel like, “Hmmm, do they think I’m schizophrenic?” (P6)

Most participants commented that they wanted the questionnaire to be individually adapted, but they also understood that this adaptation could not be perfect. Still, it was clear that simply looking at reports would not suffice to interpret the answers correctly. Elaboration and discussion was needed, and most participants seemed eager to contribute.

## Discussion

For the participants in this study, ROM/CFS triggered thoughts, emotions, and self-awareness in a complex process of ambivalent self-presentation. Through this, they could understand themselves better, feel understood by their therapist, and identify therapeutic topics. These findings provide nuance to findings from earlier studies, but also generate new and useful information about the potential of ROM/CFS.

### Understanding the results from a CFS

Answering items was an interpretative and intentional process for the participants in this study. Similarly, previous qualitative studies have described ROM/CFS as a relational process that raises awareness of inner states and feelings [13, 27, 28]. As the recent qualitative metasynthesis found [12], capturing the complexity of participants lives’ seemed challenging. Participants struggled to fit their lives and experiences into the standardized categories from the CFS. Some previous studies found that patients using ROM/CFS harbored distrust and suspicion toward service providers [29, 30]. This was not mentioned by any participants in our study. To the contrary, they all seemed cooperative and trusting. They wanted their answers to be truthful and useful, but, in line with Börjesson and Boström [13], several participants admitted to answering strategically. Though many examples were innocent (e.g., not wanting to hurt their therapist’s feelings), others were therapeutically significant (e.g., underreporting to avoid difficult subjects). It seems clear that the answers from a CFS cannot be taken as objective truths. In order to be clinically useful, the answers must be elaborated upon, with a skilled and curious therapist, and in a trusting therapeutic relationship.

### Realizing the potential of ROM/CFS

The participants in this study described a potential for ROM/CFS to raise self-awareness and reflexivity, enhance therapeutic conversations by identifying important topics, and allowing patients more influence over their therapies. This echoes the themes of collaborative practice and empowerment [12, 13, 31–35] and supports the claim that ROM can enhance therapists’ awareness and enable better adjustment to patients’ needs [3, 4]. One explanation for the variable findings on ROM/CFS efficacy could be that ROM/CFS in itself does not enhance psychotherapies. It has potential for enhancing therapeutic processes by stimulating self-awareness, reflexivity, and meaningful conversations in a trusting therapeutic relationship [12, 32], but perhaps it should also be considered a clinical skill that needs to be taught and practiced in order to be useful [36, 37].

### Limitations

Though normal for qualitative research [20], the sample size of our study requires caution in generalizing. Idiosyncrasies of the sample and the NF will have affected our findings. Previous research has shown that ROM/CFS is especially useful for patients who are deteriorating or not progressing in therapy [7–9]. Our analysis did not differentiate between demographic variables, nor therapeutic progression or outcomes. Furthermore, participants were recruited by their therapists, who may have selected patients that were progressing well, had good relationships with their therapists, and/or were using the CFS successfully. Finally, all researchers are part of a research group working with the development of the NF. Though we attempted to set all preconceptions aside, they may have affected our interpretations of the data. Despite these limitations, we believe the results from our study inform and expand upon the existing literature.

### Implications for clinical practice and further research

The findings from this study and previous research suggest that using ROM/CFS is, like psychotherapy itself, a clinical skill. For future research, this could mean extending our research questions beyond effect studies, to the how-to aspects of using different CFS. Further research would benefit from combining quantitative and qualitative methods and investigating differences between patient groups and CFS. In clinical practice, ROM/CFS should be used sensitively and flexibly, adapted to patients’ preferences and needs.

## Electronic supplementary material

Below is the link to the electronic supplementary material.

Supplementary file1 (DOCX 17 kb)

Supplementary file2 (DOCX 16 kb)

Supplementary file3 (DOCX 19 kb)
