# Supplementary material for: Complexity and potentials of clinical feedback in mental health: an in-depth study of patient processes
Source: Qual Life Res. 2020 Jun 15;30(11):3117–25. doi: 10.1007/s11136-020-02550-1 (PMC8528773; doi:10.1007/s11136-020-02550-1)
Supplement: Supplementary file 1 — Supplementary file1 (DOCX 17 kb) [file 11136_2020_2550_MOESM1_ESM.docx]

**Complexity and potentials of clinical feedback in mental health: an in-depth study of patient processes** [1]

**Online supplementary material – Interview guide** (translated from Norwegian)

Instruction

*The purpose of this interview is to access your experiences with using the clinical feedback system Norse in your treatment. We will use a video recording of the therapy session to aid your recollection, and to attain information about how the Norse is used in sessions. When, or if, we reach a point in the video where you see, or recall, something that was relevant for the interview topic, I want you to stop the video and tell me about what we witnessed. I will also ask you questions about what we just saw, and what it meant to you. After the video is finished I will ask you more general questions about your experiences with the Norse.*

*It may be uncomfortable seeing yourself on video, and it may be uncomfortable talking about your thoughts and emotions. If you feel upset, have questions or need a break, then let me know. If there are parts of the video that are irrelevant for the interview topic, or that you for other reasons want us to skip over, you may also let me know.*

*I want you to control the video, but if I see something that I find very interesting or relevant, I may also stop the video. Is there anything you would like to ask before we start the video?*

Questions for stops in the video

- What is happening here?
- How did you experience this?
- What were you thinking? What were you feeling?
- Why is this important for you?
- What’s most important about this event?
- Do you think differently about this event now than you did when it happened? If so, how?

Post-video questions:

- What is it like to answer a questionnaire before sessions?
- What do you think about the questions in the questionnaire?
- How do you experience your therapist’s use of the information from the feedback system?
- Have you been given feedback on your answers? If so, how did you experience that?
- Do you feel like the feedback system has affected your treatment? If so, how?
- Do you feel like the feedback system has affected the relationship between you and your therapist? If so, how?
- Do you feel like the feedback system has affected your life outside of treatment? If so, how?
- Do you feel like the feedback system has affected how you think of being in treatment? If so, how?
- Do you think anything about the feedback system should be changed? If so, what?

1. Solstad, S. M., Kleiven, G. S., & Moltu, C. (2020). Complexity and potentials of clinical feedback in mental health: an in-depth study of patient processes. *Quality of Life Research, 29*.
