# Supplementary material for: Complexity and potentials of clinical feedback in mental health: an in-depth study of patient processes
Source: Qual Life Res. 2020 Jun 15;30(11):3117–25. doi: 10.1007/s11136-020-02550-1 (PMC8528773; doi:10.1007/s11136-020-02550-1)
Supplement: Supplementary file 2 — Supplementary file2 (DOCX 16 kb) [file 11136_2020_2550_MOESM2_ESM.docx]

**Complexity and potentials of clinical feedback in mental health: an in-depth study of patient processes** [1]

**Online supplementary material - Adaptation of Interpersonal Process Recall for data collection**

| **Interpersonal Process Recall (IPR)** is a method for audio- or video-assisted micro-process interviews that allows for detailed descriptions of interpersonal processes [2,3]. It was first developed for instructional purposes, but has since been adopted by psychotherapy researchers because of its potential for investigating micro processes in human interactions. The processes in question are recorded by audio or video, and interviews are conducted as soon as possible thereafter, preferably within 48 hours. The recordings are used to stimulate and vivify the informants’ recollections in a manner that is not possible in conventional retrospective interviewing. The goal is to capture detailed information about their experiences before it is forgotten or merged into global post hoc perceptions.  The IPR interview starts with an introduction to the methodology and the purpose of the study (see fig. 2 in article). The recorded material is then started, and the informant is asked to stop the playback when important thoughts or experiences are evoked. At each stop, the informant is asked about their experiences in the situation, including what happened, why it was important, and what thoughts and feelings were involved. Exploration of events proceeds through the entire recording. The interview typically concludes with a debriefing to check in with the informant’s current status, and experience of the interview situation.  **References**  1. Solstad, S. M., Kleiven, G. S., & Moltu, C. (2020). Complexity and potentials of clinical feedback in mental health: an in-depth study of patient processes. *Quality of Life Research, 29*.  2. Elliott, R. (1986). Interpersonal Process Recall (IPR) as a psychotherapy process research method. In *The psychotherapeutic process: A research handbook* (pp. 503-527). New York, NY: Guilford Press; US.  3. Kagan, N. I., & Kagan, H. (1991). Interpersonal process recall. In *Practical guide to using video in the behavioral sciences* (pp. 221-230). Oxford, England: John Wiley & Sons; England. |
| --- |
