# Supplementary material for: Complexity and potentials of clinical feedback in mental health: an in-depth study of patient processes
Source: Qual Life Res. 2020 Jun 15;30(11):3117–25. doi: 10.1007/s11136-020-02550-1 (PMC8528773; doi:10.1007/s11136-020-02550-1)
Supplement: Supplementary file 3 — Supplementary file3 (DOCX 19 kb) [file 11136_2020_2550_MOESM3_ESM.docx]

**Complexity and potentials of clinical feedback in mental health: an in-depth study of patient processes** [1]

**Online supplementary material - Norse Feedback**

| **Norse Feedback (NF)** [2,3] is a clinical feedback system for routine outcome monitoring developed by the Førde Hospital Trust and standardized for the Norwegian population. Questions in NF were generated from clinicians’ and patients’ needs [2] and tested and refined in clinical implementations [4,5] in ordinary settings. NF uses digital person-adaptive computer technology to combine the advantages of standardized measures with patient and clinician preferences for idiographic approaches [6]. This method for measure development is described [7] and psychometrics properties of the NF are reported [8] alongside this paper. Aiming to make NF available for a broad range of patients has been a priority in development and research [5]. The system is administered electronically via personal computer, computer tablet, or mobile phone. Patients may use computer tablets at the outpatient clinic if they prefer to answer the NF there. Hospitalized patients who are unable to answer the NF themselves are assisted by clinical staff. In a standard schedule, patients will answer the NF in advance of each session, typically weekly or biweekly. For hospitalized patients, the NF is administered once a week.  NF currently consists of a maximum total of 99 items loading onto multiple scales concerning common psychiatric symptoms, alcohol and drug abuse, medications, social and personal functioning, strengths and resources, therapeutic needs, therapeutic progress, and the therapeutic alliance. At intake, before their first session, patients are given all items apart from those concerning therapeutic needs, progress and alliance. The latter are excluded at intake because the patient has not yet met their therapist, and are included from the second administration onwards. Patients answer by indicating their agreement to the items on a seven point Likert scale. Subsequent administrations learn from the patients’ initial responses by comparing scores to risk adjusted norms to evaluate which areas are of particular relevance for any individual’s status and process. On scales where the patient reports below an empirical threshold, the number of items is automatically reduced to one trigger item, which is the one providing most information at moderate difficulty in item response theory analyses [8]. On scales where the patient reports distress above a threshold, all items are activated, for increased measure precision. If a scale has already been collapsed to the trigger item, the original items are expanded if the trigger item is answered above an empirical threshold.  The NF generates a visual report that summarizes the patient’s development throughout treatment. Therapists can also access the full list of patients’ responses to each item. The visual report includes information about suicidality, flagging scores above the clinical threshold. The report is available to the therapist immediately after the questionnaire is completed, but not to the patient. Therapists may provide feedback to patients about their scores, for example by printing the results out or displaying them on a computer screen.  **References**  1. Solstad, S. M., Kleiven, G. S., & Moltu, C. (2020). Complexity and potentials of clinical feedback in mental health: an in-depth study of patient processes. *Quality of Life Research, 29*.  2. Moltu, C., Veseth, M., Stefansen, J., Nøtnes, J. C., Skjølberg, Å., Binder, P.-E., et al. (2018). This is what I need a clinical feedback system to do for me: A qualitative inquiry into therapists’ and patients’ perspectives. *Psychotherapy Research, 28*(2), 250-263, doi:10.1080/10503307.2016.1189619.  3. Norse Feedback (2019). Norse Feedback homepage. <www.norsefeedback.no>. Accessed 10. october 2019.  4. Hovland, R. T., & Moltu, C. (2019). Making way for a clinical feedback system in the narrow space between sessions: navigating competing demands in complex healthcare settings. *International Journal of Mental Health Systems, 13*(1), 68, doi:10.1186/s13033-019-0324-5.  5. Hovland, R. T., & Moltu, C. (2019). The challenges of making clinical feedback in psychotherapy benefit all users: A qualitative study. *Nordic Psychology*, 1-15, doi:10.1080/19012276.2019.1684348.  6. Jensen-Doss, A., Smith, A. M., Becker-Haimes, E. M., Mora Ringle, V., Walsh, L. M., Nanda, M., et al. (2018). Individualized Progress Measures Are More Acceptable to Clinicians Than Standardized Measures: Results of a National Survey. *Administration and Policy in Mental Health and Mental Health Services Research, 45*(3), 392-403, doi:10.1007/s10488-017-0833-y.  7. Nordberg, S., McAleavey, A. A., & Moltu, C. (Submitted for Review). Continuous quality improvement in measure development: Lessons from building a novel clinical feedback system. *Quality of Life Research*.  8. McAleavey, A. A., Nordberg, S., & Moltu, C. (Submitted for review). Initial quantitative development of the Norse Feedback system: A novel adaptive multidimensional tool for use in routine mental healthcare. *Quality of Life Research*. |
| --- |
